# Harris RJ, Korolchuk VI, eds. Biochemistry and Cell Biology of Aging: Part I Biomedical Science: Subcellular Biochemistry; Springer; e-book 531 pages, 2018; ISBN 978-981-13-2834-3; eISBN 978-981-13-2835-0

**DOI:** 10.3325/cmj.2020.61.205

**Published:** 2020-04

**Authors:** Koraljka Gall Trošelj

**Field of Medicine:** Biomedicine; biogerontology

**Audience:** Biomedical scientists, students of medicine

**Purpose:** To comprehensively present various aspects of aging, aging-associated diseases, and potential modes of therapeutic intervention.

This book is divided into 17 chapters. The first chapter discusses the current state of the art with respect to the free radical theory of aging and the role of reactive oxygen species (ROS) and antioxidants (endogenous and exogenous; natural and synthetic) in the process of aging. The authors critically present the differences between rodent and human models and provide possible reasons for non-comparable data obtained in these models, especially in the context of reductive stress. They also question the (uncritical) use of various antioxidants and state that the key to successful life-span extension is stimulating DNA damage response pathways and modulating mitochondrial function. In chapter 14, the free radical theory of aging is further discussed in the context of the roles of various enzymes involved in ROS metabolism in aging. The data are presented in a very interesting way and from several points of view, including various biological models that have been traditionally used for studying aging.

The second chapter is very informative for those who want to learn the basics of autophagy, particularly in aging. It also introduces some basic facts on cellular senescence and its functional connection to another mark of aging – an aberrant inflammatory response. These phenomena are further discussed in the context of diseases affecting older populations: Alzheimer’s disease, macular degeneration, and cancer. Finally, the authors comprehensively reviewed the current approaches/recommendations for stimulating autophagy in order to slow the aging process and increase longevity (calorie restriction, sunlight exposure, exercising). Although not elaborated in detail, the molecular genetics part of this chapter makes a good introduction to the third chapter, which primarily deals with the role of IGF-1 and mTOR in aging and age-related disease in terms of nutrient sensing and signaling. It specifically discusses the possibility of intervention, while critically pointing to the paradoxical scenarios that occur with respect to IGF-1 roles in some human pathologies: neurodegenerative diseases, age-related bone loss, metabolic syndrome, and obesity. This chapter is a must read especially for those who, for whichever reason, tend to simplify the age-related processes. It recognizes and correctly addresses some critical issues in the field, eg, the unrepeated studies identifying intervention strategies of unclear validity, the need for tissue- and context-specific animal models, and cautious interpretation of experimental findings.

The fourth chapter gives a very informative historical overview of the concept of aging and also discusses the impact of some aspects of cholesterol metabolism on aging. The authors suggest that ROS can increase the rate of cholesterol biosynthesis and present several mathematical models that represent various aspects of cholesterol metabolism.

The excellently written fifth chapter explains the signaling cascades responsible for the decline of stem cell function in aging. This chapter requires considerable knowledge about cell type-specific signaling pathways related to the transforming growth factor beta, NOTCH, WNT/beta-catenin, JAK/STAT, and MAPK/ERK. Absolute beginners who want to learn about these signaling pathways need to read each sentence carefully and study the cited references. The content of this chapter is logically extended in the 11th chapter, which explains signal transduction pathways in aging. It primarily focuses on pro-longevity interventions associated with insulin/IGF-1-like signaling, mTOR, RAS/MAPK, and AMPK-mediated signaling. The intersections and specific feedback mechanisms of these pathways in aging are explained in detail. This chapter requires a high level of pre-existing knowledge about molecular players included in these pathways. The authors illustrate the role of NRF2 activity in longevity and aging from the point of aberrant signal transduction, which can be the target for therapeutic intervention (rapamycin, metformin).

The sixth chapter explains the importance of creatine and creatine kinase activity in aging. This very informatively written chapter brings together diverse information and data. The authors explain the relationship between creatine depletion and the phenotypes of motor and cognitive impairments/dysfunctions as the hallmarks of aging process. They critically point to the fact that, although short-term creatine supplementation may be of benefit for some conditions, its effect on neurodegenerative diseases and age-associated declines needs to be further studied. The complex role of vitamin D in aging (chapter eight) is described from various perspectives. These include the very interesting epidemiological studies on vitamin D supplementation and calcium metabolism, with explanation for the possible differences obtained in these studies. Those not deeply involved in this field would be surprised to learn that there is still no consensus on circulating 25(OH)D concentrations used to define vitamin D status, although there is no doubt that 17% to 58% of older Europeans are vitamin D deficient (defined as circulating 25(OH)D less than 30 nmol/L). Chapter 15 is dedicated to B vitamins and aging, recognizing the reasons for B vitamin deficiency in an aging population: low intake (B2 and B9), malabsorption (B12), and increased requirement (B6), which are commonly combined with drug-nutrient interactions, genetic disorders, and certain medical conditions. Specific attention was given to B1 and neurodegeneration mechanism, B2 and oxidative damage, B3 and its roles (as a precursor of NAD/NADP) in mitochondria maintenance, B4 and Alzheimer's disease, B5 and longevity in general, B6 and glycation, B9 in stroke and neurodegenerative diseases, and B12 and depression in elderly patients.

The seventh chapter discusses the importance of collagen and elastin for the formation of extracellular matrix and its change during aging. For those who are not familiar with matrix stiffening with increasing age – age-associated collagen glycation and formation of advanced glycation end products and protein carbamylation, this chapter, although informative, is anything but easy to read. It is a pity that the seventh chapter is not preceded by a chapter covering the most important topics in glycobiology and aging (17), as this would make reading far easier.

Those who want to learn about the most recent discoveries in the field of telomeres, telomerases, and telomeric interactome in the context of aging, must read the chapter nine. It provides hard-core evidence on multilevel connections between telomere integrity and DNA repair pathways. It also describes complex molecular mechanisms responsible for the induction of senescence by dysfunctional telomeres. The regulatory mechanisms involved in telomerase expression, activity, and way of acting are particularly well described. However, to be able to follow the chapter one should have a very solid background in molecular biology. The specific role of telomeres and telomerase activity during aging is described in detail in immune cells, and further explained in aging, based on population studies, as well as in reproductive aging. Intriguingly so, the authors discuss the complex background responsible for different rates of telomere shortening, which can depend on genetic make-up, including, but not limited to, the expression of antioxidant enzymes, nutrition, life history factors, and inflammation. As a logical extension, possible lifestyle interventions on the telomeres complex are thoroughly discussed. Chapter 10 describes the consequences of genome instability in the context of DNA repair mechanisms and the DNA damage response with respect to developmental abnormalities, accelerated aging, and cancer. These repair mechanisms are also discussed in the context of calorie restriction intervention.

Chapters 12 and 13 form a functional unit that presents various functional links between the gut microbiome (12), nutrition (13), and aging. The authors explain the development of age-associated changes in the gut microbiota, and how these changes resemble those associated with dysbiosis in terms of marks of inflammation, which is a known risk factor for the progression of several age-related diseases. The concept of the nine hallmarks of aging (some of them addressed in previously described chapters, ie, cellular senescence, genomic stability, telomere attrition, mitochondrial function) was used as the baseline for discussing the influence of diet on the hallmarks of aging, with a focus on the beneficial effects of dietary restriction and the Mediterranean diet. Additionally, chapter 15 provides a good overview of interventional studies on humans, and discusses the influence of diet and dietary interventions on the epigenome.

Chapter 16 is dedicated to epigenetic changes associated with aging from the perspectives of global alterations in gene expression and non-coding RNAs. The basic epigenetic mechanisms – DNA methylation and histone modifications – were explored in the context of specific spatial redistribution of heterochromatin associated with senescence and aging.

**Highlights:** This book explores many interesting yet different aspects of aging. In some parts, it offers a valid recapitulation of basic physiology (synthesis and metabolism of vitamin D), while in other it presents complex sets of data that are logically connected – from the basic cellular signaling pathway to the epidemiological interventional study. However, for understanding all the connections offered, the reader should approach this material with patience and curiosity.

**Limitations:** This is not material for beginners, unless they want to invest considerable time and effort to understand all that is written. It is not easy to follow the parts on basic signaling pathways, which, condensed as they are, have some species-associated specifics. Most of the chapters present data obtained in various biological models (yeast, nematodes, rodents) and not necessarily humans. It may become overwhelming and confusing if one wants to read about molecular events and signaling pathways relevant for interventional studies on humans. One may argue that there is redundancy with respect to some signaling pathways, but I do not see it as a limitation. Quite the contrary, the strength of these pathways varies among different organs and, seemingly so, determines the individual response to the process of aging.

**Commentary:** Although the book composition does not allow for a smooth flow of the chapters, which are not uniformly written, it represents a logical unit filled with valuable information worth reading and further studying. In this broad spectrum of approaches to aging, I believe that readers will find their favorite chapters, as I found mine.

